# Research on methods for estimating reference crop evapotranspiration under incomplete meteorological indicators

**DOI:** 10.3389/fpls.2024.1354913

**Published:** 2024-07-08

**Authors:** Xuguang Sun, Baoyuan Zhang, Menglei Dai, Ruocheng Gao, Cuijiao Jing, Kai Ma, Shubo Gu, Limin Gu, Wenchao Zhen, Xiaohe Gu

**Affiliations:** ^1^ College of Agronomy, Hebei Agricultural University, Baoding, Hebei, China; ^2^ Research Center of Information Technology, Beijing Academy of Agriculture and Forestry Sciences, Beijing, China; ^3^ State Key Laboratory of North China Crop Improvement and Regulation, Baoding, Hebei, China; ^4^ State Key Laboratory of Wheat Improvement and College of Agronomy, Shandong Agricultural University, Taian, Shandong, China; ^5^ Key Laboratory of North China Water-saving Agriculture, Ministry of Agriculture and Rural Affairs, Baoding, Hebei, China

**Keywords:** reference crop evapotranspiration, Penman-Monteith, FAO-24 radiation, meteorological indicators, Bayesian estimation

## Abstract

**Background:**

Accurate estimation of reference crop evapotranspiration (ET_0_) is crucial for farmland hydrology, crop water requirements, and precision irrigation decisions. The Penman-Monteith (PM) model has high accuracy in estimating ET_0_, but it requires many uncommon meteorological data inputs. Therefore, an ideal method is needed that minimizes the number of input data variables without compromising estimation accuracy. This study aims to analyze the performance of various methods for estimating ET_0_ in the absence of some meteorological indicators. The Penman-Monteith (PM) model, known for its high accuracy in ET_0_ estimation, served as the standard value under conditions of adequate meteorological indicators. Comparative analyses were conducted for the Priestley-Taylor (PT), Hargreaves (H-A), McCloud (M-C), and FAO-24 Radiation (F-R) models. The Bayesian estimation method was used to improve the ET estimation model.

**Results:**

Results indicate that, compared to the PM model, the F-R model performed best with inadequate meteorological indicators. It demonstrates higher average correlation coefficients (R^2^) at daily, monthly, and 10-day scales: 0.841, 0.937, and 0.914, respectively. The corresponding root mean square errors (RMSE) are 1.745, 1.329, and 1.423, and mean absolute errors (MAE) are 1.340, 1.159, and 1.196, with Willmott's Index (WI) values of 0.843, 0.862, and 0.859. Following Bayesian correction, R^2^ values remained unchanged, but significant reductions in RMSE were observed, with average reductions of 15.81%, 29.51%, and 24.66% at daily, monthly, and 10-day scales, respectively. Likewise, MAE decreased significantly, with average reductions of 19.04%, 34.47%, and 28.52%, respectively, and WI showed improvement, with average increases of 5.49%, 8.48%, and 10.78%, respectively.

**Conclusion:**

Therefore, the F-R model, enhanced by the Bayesian estimation method, significantly enhances the estimation accuracy of ET_0_ in the absence of some meteorological indicators.

## 1 Introduction

Agriculture stands as the largest consumer of freshwater (Food, and Nations, A. O. o. t. U, 2017; Boretti and Rosa, 2019). Efficient freshwater resource utilization in agricultural product production is a pivotal concern for sustainable development (Tunalı et al., 2023). Particularly in arid or semiarid climates, irrigation plays a critical role in food production systems and economies. However, limited available water may not meet the demands of food production, necessitating effective scheduling methods to optimize crop yields with constrained water resources (King et al., 2020; Gu et al., 2021; Zhang et al., 2021). There is a growing emphasis on enhancing water productivity by improving evapotranspiration (ET) efficiency in food production (Xu et al., 2018; Qiu et al., 2021). This shift toward sustainable and efficient water use in agricultural systems underscores the need for precise estimations of crop transpiration and soil ET (Yong et al., 2023a).

Accurate estimation of crop ET is instrumental in on-farm irrigation management, facilitating improvements in irrigation practices and systems (Nyolei et al., 2021). This enhances water productivity, enabling more farmers to derive benefits from limited water resources and achieve increased food production (Perry et al., 2009; Akumaga and Alderman, 2019). Crop water requirement holds a pivotal role in the farm water cycling system. Modern water-saving irrigation theory advocates for deficit-regulated irrigation based on crop water requirement. This approach maximizes yields while maintaining optimal water levels in the root zone and minimizing nutrient losses, disease susceptibility, and operating costs (Tunalı et al., 2023). Reference crop evapotranspiration (ET_0_) forms the basis for calculating crop water requirements. Over nearly a century, the estimation methods for ET_0_ have been extensively studied globally. Although lysimeters are one of the most accurate tools for direct calculation of ET_0_, they are not suitable for this purpose due to their relatively higher cost, the time required for the complex measurements, and their limited accessibility at most sites (Chia et al., 2020). Another common strategy is to calculate ET_0_ indirectly using experimental formulae and meteorological factors (Salam and Islam, 2020). The Penman-Monteith model, widely utilized, comprehensively describes ET processes, incorporating meteorological and vegetation physiological characteristics (Monteith, 1965). This model estimates ET as water vapor diffusing from the canopy surface through aerodynamic and gradient methods (Monteith and Unsworth, 2013). Although the ET_0_ obtained by the PM model is reliable, it faces limitations due to the stringent requirements for climate data at specific locations (Alam et al., 2024).The Priestley-Taylor (PT) model, a radiation-based approach, calculates actual evapotranspiration using an empirically derived potential ET coefficient α (Kohler et al., 1955). This model minimizes differences in land cover and soil moisture (Priestley and Taylor, 1972). Hargreaves and Samani (1985) introduced the Hargreaves (H-A) model, utilizing maximum and minimum temperatures and extraterrestrial radiation to estimate ET_0_. Recognized for its simplicity and accuracy, the H-A model is considered one of the most reliable methods for ET_0_ estimation (Jensen et al., 1997). The Mc-Cloud method, relying on average daily air temperature, treats potential ET as an exponential function of temperature. This method is particularly suitable for regions with large temperature variations (Valipour, 2015). The FAO-24 Radiation method, derived from the Makkink formula, exhibits variable accuracy based on altitude (Hauser et al., 1999). Each of these methods contributes to the rich landscape of ET_0_ estimation, offering diverse options for addressing the complexities of agricultural water management.

The Penman-Monteith (PM) model has demonstrated applicability to various surfaces across diverse spatial and temporal scales (Allen et al., 2006; Matejka et al., 2009). In order to exclude the impact of climate change on reference evapotranspiration (ET_0_), it is necessary to fully consider the impact of different annual rainfall on the evapotranspiration model. Therefore, it is necessary to select a representative hydrological year to verify the model to reflect the universality of the model (Yong et al., 2023b; Latrech et al., 2024). It is recommended as the standard method for estimating ET_0_ and serves as a benchmark for validating other evapotranspiration models (Allen, 1998). The PM method exhibits versatility across environments and climates, eliminating the need for local calibration. Extensive validation in various climates, including the use of lysimeter facility, supports its reliability (Landeras et al., 2008; Shiri et al., 2012). Reference evapotranspiration relies on meteorological factors such as radiation, air temperature, humidity, and wind speed, with temperature being the most influential. The PM model, chosen as the standard method for ET_0_ estimation, requires daily maximum and minimum temperatures, relative humidity, solar radiation, and wind speeds (Luo et al., 2014). However, a notable limitation of PM models is their demand for an extensive array of uncommon meteorological data, including relative humidity, solar radiation, and wind speed (Droogers and Allen, 2002; Almorox et al., 2015). In the absence of comprehensive meteorological information, accurately calculating ET_0_ using PM models becomes challenging (Feng et al., 2017). Public weather forecasts typically include only weather conditions, maximum and minimum temperatures, wind levels, and wind directions. To address this, four widely used ET_0_ estimation models with lower meteorological data requirements have gained prominence. The PT model omits the need for wind speed and humidity data, the H-A model calculates ET_0_ based on temperature and solar radiation, the M-C model simplifies ET_0_ calculation based on temperature, and the F-R model primarily uses sunshine duration data. An ideal ET_0_ estimation method should minimize the number of required meteorological variables without compromising accuracy (Shih, 1984; Traore et al., 2010). Recent studies (Choi et al., 2018; Gao et al., 2021; Yamaç, 2021; Dimitriadou and Nikolakopoulos, 2022; Elbeltagi et al., 2022) have achieved superior ET_0_ estimation results compared with traditional methods with limited climate data. As a result, there is a pressing need to comprehend the temporal distribution of crop ET and anticipate its future changes using constrained meteorological information.

In the current study, the calculation of ET_0_ is based on the PM model with more meteorological data, or the model with less meteorological data to blur the calculation, but the accuracy is not high. Therefore, in order to accurately calculate ET_0_ to successfully monitor crop water requirements and prevent excessive or insufficient irrigation. The primary aim of this study is to conduct a comparative analysis of different ET_0_ estimation models under conditions of incomplete meteorological indicators. Additionally, the study seeks to enhance the optimal estimation model to better suit the requirements for ET_0_ estimation in the presence of insufficient meteorological data. The most important studies are listed below:

1) Conduct a comparative performance analysis of the PM model and four alternative ET_0_ calculation models (H-A, PT, F-R, and M-C), which require fewer meteorological data inputs. Evaluate their effectiveness in estimating ET across various hydrologic years.
2) Investigate and identify a simplified method for calculating ET_0_ distinct from the PM model. Explore alternative models or approaches that offer simplicity while maintaining accuracy in ET_0_ estimation.
3) Employ Bayesian estimation to rectify the empirical parameters of the optimal ET estimation model.

## 2 Materials and methods

### 2.1 Overview of the study area

**Figure 1:**
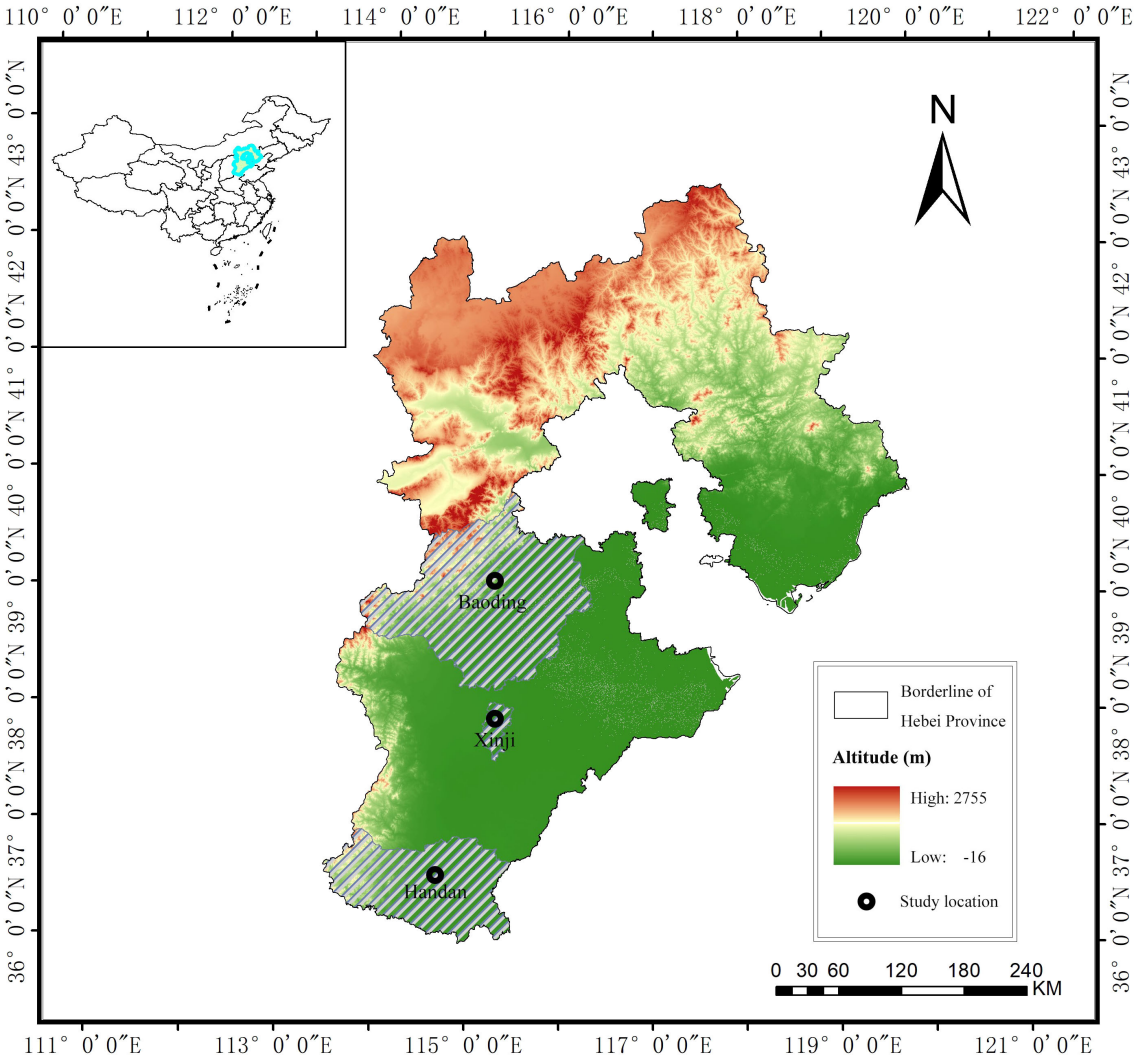
Study area.

The Haihe Plain (34°48′–41°3′N, 112°33′–119°50′E), situated in the northern part of the North China Plain, encompasses the plain areas of Beijing, Tianjin, and Hebei, as well as the northern regions of Henan and Shandong Provinces (**Figure 1**). Renowned as a primary grain-producing region, our study specifically focuses on the large and medium-sized cities of Baoding, Xinji, and Handan within the plain part of Hebei Province. The climate of the Haihe Plain is characterized by a temperate semi-humid and semiarid continental monsoon climate. This climate exhibits four distinct seasons, featuring a dry and windy spring, a hot and rainy summer, a mild and cool autumn with slightly more cloudiness and rain in early autumn, and a cold winter with minimal rain and snow. These pronounced seasonal variations contribute to noticeable changes in ET_0_ within the study area.

### 2.2 Data preparation

The study is conducted in Baoding, Xinji, and Handan cities in Hebei Province, China. Meteorological data were sourced from the Meteorological Information Center of the National Meteorological Administration (http://www.nmic.cn/). The time span covered by the meteorological data is 1991–2019 for Baoding, 2000–2021 for Xinji, and 1991–2019 for Handan. The comprehensive meteorological datasets encompass information such as station name, elevation of the meteorological station, observation time, mean barometric pressure, mean water vapor pressure, mean air temperature, daily maximum temperature, daily minimum temperature, mean relative humidity, 8–8-h rainfall (24-h cumulative rainfall from 8 a.m. to 8 a.m. the next day), mean wind speed, and sunshine hours.

### 2.3 Selection of typical hydrological years

To mitigate the impact of annual rainfall variations on ET model estimation, a specific hydrological year was carefully chosen for the test area. Separate validations were conducted for each identified typical hydrological year to uphold model accuracy. The selection of typical hydrological years followed a process whereby cumulative annual rainfall data for Baoding City (1991–2019), Xinji City (2000–2021), and Handan City (1991–2019) underwent frequency exclusion. The annual rainfall was then ranked in descending order, and cumulative frequencies were computed for accurate year selection.


(1) p=m/(N+1)

where p represents the cumulative frequency, m is the ordinal number of years for rainfall after the treatment of rainfall frequency ranking, and N is the total number of years of rainfall. Utilizing the Pearson Type III curve for fitting, the rainfall values corresponding to p = 25%, 50%, 75%, and 90% are typically considered as the design values for high flow, median water, low flow, and special dry years.

### 2.4 ET_0_ calculation method

The Haihe Plain region experiences four distinct seasons, marked by significant climatic variations. To assess the calculation accuracy of different ET_0_ models during each fertility period of crops, daily ET_0_ values for each identified typical hydrological year were computed using five ET_0_ models, calculating daily ET_0_ values for each typical hydrological year using five ET_0_ models, and further obtaining monthly and 10-day ET_0_ values.

(1) The Penman-Monteith model

The meteorological data utilized in the model encompass insolation, radiation, temperature, humidity, and wind speed. The Penman-Monteith equations are formulated to accurately predict ET_0_ across diverse locations and climatic conditions, although they exhibit high demands for meteorological data. Previous studies applied the Penman-Monteith model in controlled environments (Doorenbos, 1977; Smith et al., 1991), emphasizing the importance of determining evaporative losses in the presence of various natural and anthropogenic land cover interventions. This approach aids in identifying the contributors to evaporative losses. The FAO Penman-Monteith model employed in this study is derived from the original Penman-Monteith equation, aerodynamic drag equation, and surface drag equation, as follows:


(2) $$ET_{0}=\frac{0.408\Delta \left(R_{n}-G\right)+\gamma \frac{900}{T+273}u_{2}\left(e_{s}-e_{a}\right)}{\Delta +\gamma \left(1+0.34u_{2}\right)}$$

where R_n_ is the net radiation at the crop surface (MJ m^-2^ day^-1^), G is the soil heat flux (MJ m^−2^ day^−1^), T is the air temperature at a height of 2 m (°C), u_2_ is the wind speed at a height of 2 m (ms^−1^), e_s_ is the saturated water vapor pressure (kPa), e_a_ is the actual water vapor pressure (kPa), e_s_ − e_a_ is the difference in saturated water vapor pressure (kPa), Δ is the slope vapor pressure curve (kPa °C^−1^), and γ is the psychrometric constant (kPa °C^−1^).

(2) The Priestly-Taylor model

The meteorological data utilized in the model consist of insolation, radiation, and temperature (Priestley and Taylor, 1972). The PT model establishes a relationship between heat flux and evaporation. Notably simpler than the PM model, it eliminates the need for wind speed and humidity data, rendering it more convenient for application over large areas. However, subsequent studies have indicated that the PT model is better suited for humid areas (Priestley and Taylor, 1972; Pereira et al., 2007) and may not perform as well in arid regions. The formula is as follows:


(3) $$ET_{0}=\alpha \frac{\text{Δ}}{\Delta +\gamma }\left(R_{n}-G\right)\;$$

where α coefficient is mainly considered the influence of aerodynamic factors, in general, taking 1.26; Δ is the slope vapor pressure curve (kPa °C^−1^), γ is the psychrometric constant (kPa °C^−1^), R_n_ is the net radiation on the surface of the crop (MJ m^−2^ day^−1^), and G is the heat flux of the soil (MJ m^−2^ day^−1^).

(3) The Hargreaves model

The Hargreaves model, introduced by Hargreaves and Samani (Hargreaves and Samani, 1985), simplifies the estimation of ET_0_. This model necessitates only the average daily maximum and minimum temperatures along with solar zenith radiation (Hargreaves and Allen, 2003), thereby reducing the need for extensive raw data. This characteristic makes it feasible to utilize observations for estimating ET_0_ in regions where meteorological data are limited. The formula is as follows:


(4) $$ET_{0}=C_{0}R_{a}\left(T_{mean}+17.8\right)\sqrt{T_{max}-T_{min}}$$

where T_mean_, T_max_, and T_min_ represent the daily mean, daily maximum, and daily minimum temperatures, respectively; R_a_ is the atmospheric upper boundary solar radiation; and C_0_ is the conversion factor, taken as 0.0023.

(4) The Mc-Cloud model

The Mc-Cloud model, introduced by McCloud in 1955, offers a simplified equation for estimating ET_0_ based solely on temperature (McCloud, 1955). The formula is as follows:


(5) $$ET_{0}=KW^{1.8T_{mean}}$$

where K and W are constant terms, 0.254 and 1.07, respectively, and T_mean_ is the average temperature, °C.

(5) The FAO-24 Radiation model

The FAO-24 Radiation model, derived from the Makkink formula (Hauser et al., 1999), calculates ET_0_ exclusively from solar radiation data. The formula is as follows:


(6) $$\;ET_{0}=a+b\left(\frac{\Delta }{\Delta +\Upsilon }R_{s}\right)\;$$

where a and b are empirical coefficients with values of 0.18 and 0.50, respectively; Δ is the slope vapor pressure curve (kPa °C^−1^); γ is the psychrometric constant (kPa °C^−1^), and R_s_ is the incoming short wave solar radiation, (MJ·m^−2^·day^−1^).

### 2.5 Modifying evapotranspiration models using Bayesian estimation

The ET_0_ values for each typical hydrological year were computed using the aforementioned five ET_0_ models (Equations 2–6). Simulated values from the PM model served as the standard for analyzing the performance of the H-A, PT, F-R, and M-C models. The objective is to identify the most suitable and recommended model for simplified ET_0_ estimation in the Haihe Plain region. Employing Bayesian theory, which involves both prior and posterior distributions, possible outcomes were obtained by reestimating the probability of an event occurring based on estimates of existing data. Bayesian estimation was iteratively applied to infer the model parameters, correcting the empirical parameters of the ET model. This iterative process enhances the model’s adaptability and accuracy in the study area.

### 2.6 Model performance statistics

Utilizing the original eight meteorological data inputs (daily minimum temperature, daily maximum temperature, daily average temperature, geographic latitude and longitude, altitude, average relative humidity, actual sunshine duration, and wind speed), the ET_0_ inputs of the PM model were selected as the model’s calibration values. Statistical measures, including the R^2^, RMSE, MAE, and WI (Equations 7–10) were employed as key factors for evaluating the model. These evaluation metrics are calculated as follows:


(7) $$R^{2}={\left[\frac{\sum_{i=1}^{N}\left(P_{i}-\bar{P}\right)\left(Q_{i}-\bar{Q}\right)}{\sqrt{\sum_{i=1}^{N}{\left(P_{i}-\bar{P}\right)}^{2}}-\sqrt{\sum_{i=1}^{N}{\left(Q_{i}-\bar{Q}\right)}^{2}}}\right]}^{2}$$

(8) $$RMSE=\sqrt{\frac{1}{N}\sum_{i=1}^{N}{\left(P_{i}-Q_{i}\right)}^{2}}$$

(9) $$MAE=\frac{1}{N}\sum_{i=1}^{N}\left|P_{i}-Q_{i}\right|$$

(10) $$\text{WI}=1-\frac{\sum_{i=1}^{N}{\left(Q_{i}-P_{i}\right)}^{2}}{\sum_{i=1}^{N}{\left(\left|Q_{i}-\bar{P}\right|+\left|P_{i}-\bar{P}\right|\right)}^{2}}$$

where N is the number of data series; P_i_ and Q_i_ (mm/d) are the simulated and PM model ET_0_ values, respectively; and $\bar{P}$ and $\bar{Q}$ (mm/day) are the average of the simulated and PM model ET_0_ values, respectively.

## 3 Results and analysis

### 3.1 Selection of hydrological year

**Figure 2:**
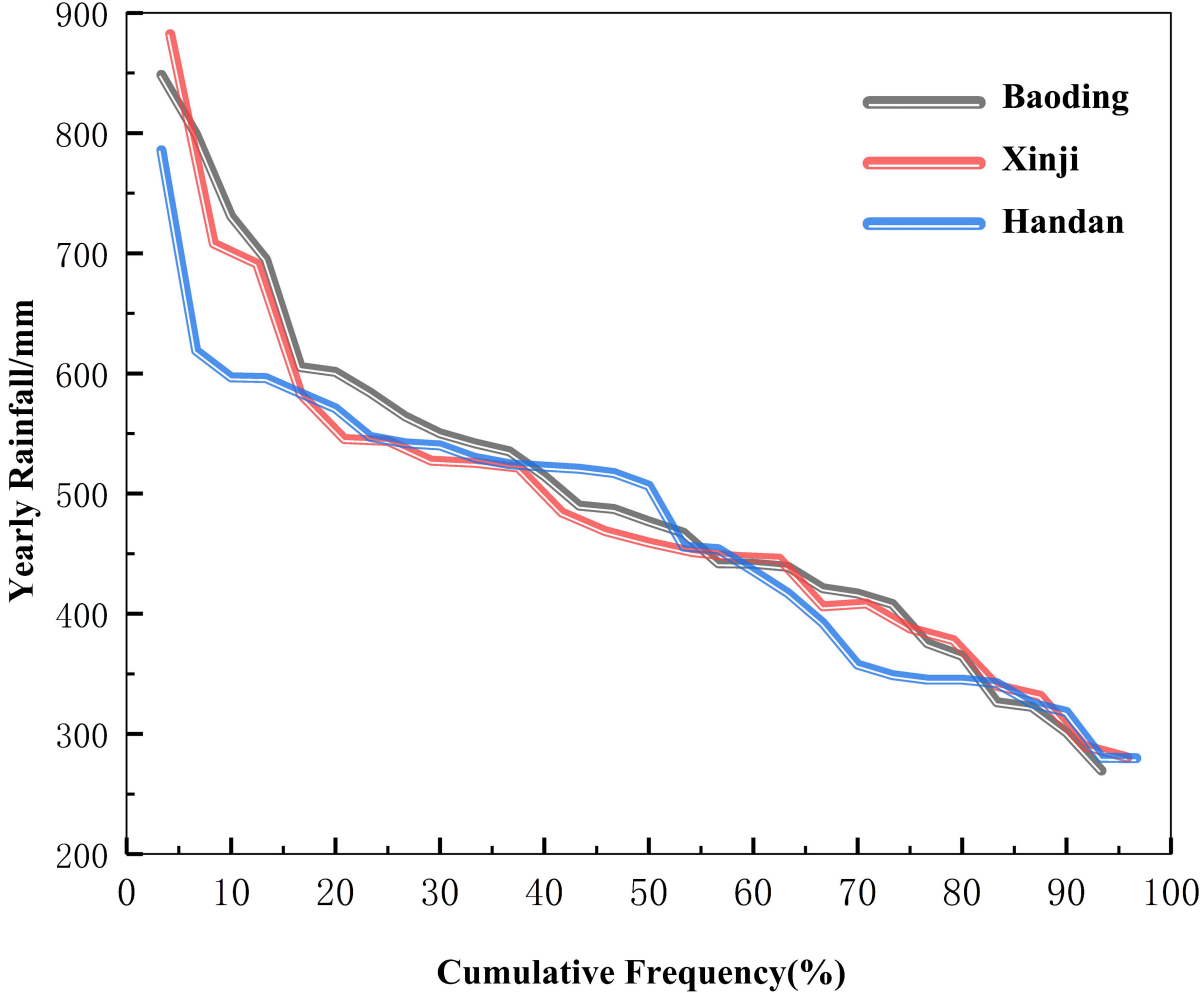
Typical hydrological year selection.

Based on the rainfall data from Baoding (1991–2019), Xinji (2000–2021), and Handan (1991–2019), the selection of typical hydrological years was carried out sequentially using Equation 1. The identified years for Baoding are 2008, 2009, 1992, and 1997, representing the high flow year (p = 25%), median water year (p = 50%), low flow year (p = 75%), and special dry year (p = 90%), respectively. Similarly, for Xinji, the years are 2004, 2010, 2007, and 2006, and for Handan, the years are 1993, 2014, 2006, and 2017, corresponding to the same hydrological conditions (**Figure 2** and **Table 1**).

**Table 1:** Selection of typical hydrological years in some areas of the Haihe Plain region.

Study area	Year	Annual precipitation	Cumulative frequency	Hydrological year type
Baoding	2008	564.3	25%	High flow year
	2009	476.9	50%	Median water year
	1992	375.4	75%	Low flow year
	1997	301.3	90%	Special dry year
Xinji	2004	543.9	25%	High flow year
	2010	459.3	50%	Median water year
	2007	387.8	75%	Low flow year
	2006	290.2	90%	Special dry year
Handan	1993	541.9	25%	High flow year
	2014	506.6	50%	Median water year
	2006	345.2	75%	Low flow year
	2017	318.2	90%	Special dry year

### 3.2 Comparative analysis of daily ET_0_ values for different typical hydrologic years

**Figure 3:**
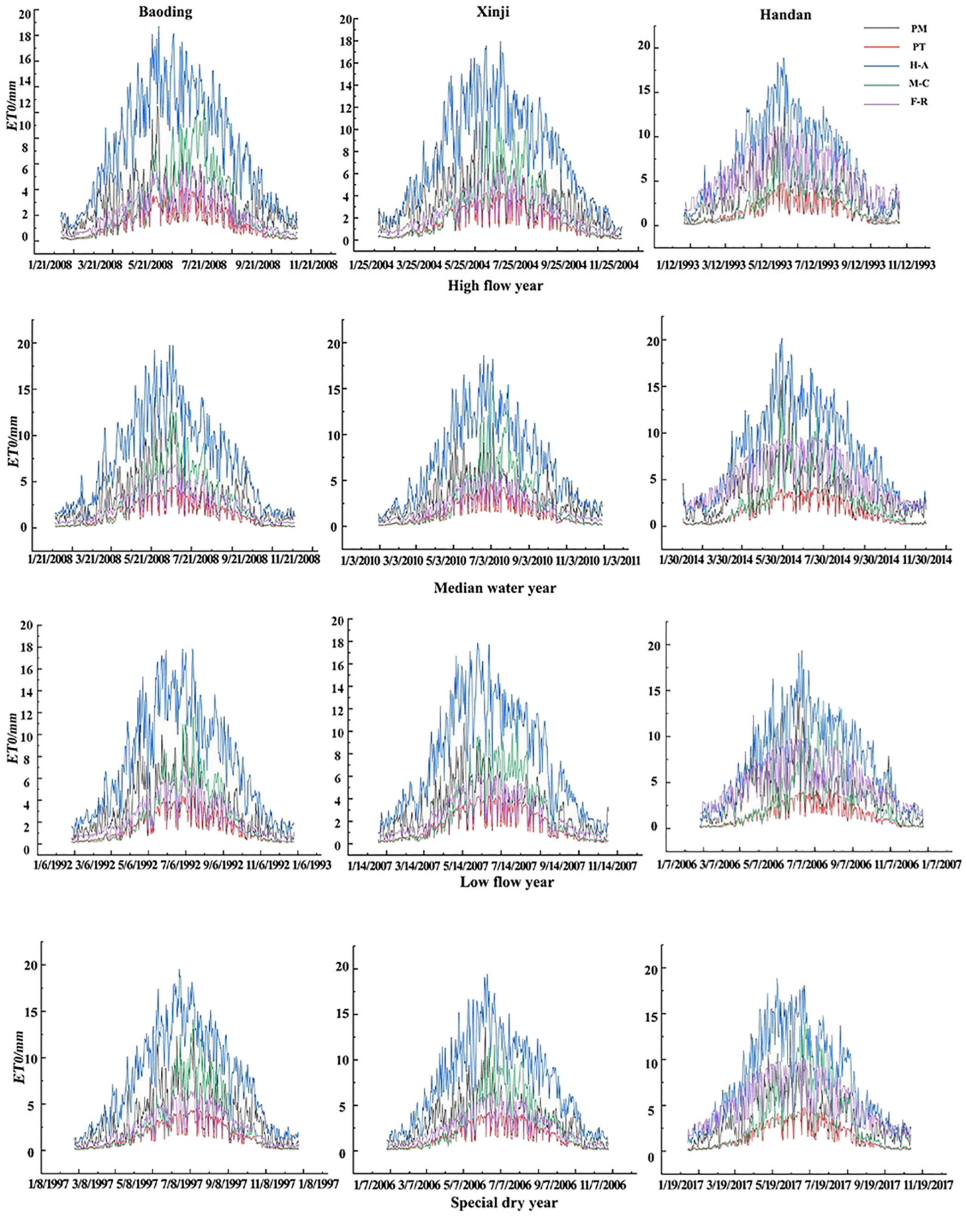
ET_0_ values of different models under typical hydrological year at daily scale.

In **Figure 3**, the day-by-day ET_0_ trends of the five models across the three regions under various typical hydrological years exhibit patterns approximating monotonically increasing and decreasing parabolas. The upward segment spans from January to July, followed by a downward segment from July to December, with peak values occurring in the months of June and July for all five models. Comparatively, the H-A model consistently produces higher ET_0_ results than the PM model throughout the year. In contrast, the PT and F-R models consistently yield lower ET_0_ results than the PM model throughout the year. The M-C model produces higher ET_0_ results than the PM model in the months of June–September but lower values in the remaining months.

Using the PM model-calculated ET_0_ values as the standard, a comparative analysis of daily ET_0_ values for the remaining four ET models is conducted under different typical hydrological years. At the daily scale, the H-A model yielded slightly larger results than the PM model while the F-R and PT models produced slightly lower values. The remaining three models showed relatively close results to the PM model, except for the H-A model. The PM model and the other four models were used to calculate the RMSE, MAE, and R^2^ for each typical hydrological year. The results are presented in **Table 2**. At a significance level of 0.01, the PT, H-A, and F-R models exhibited good correlation with the PM model’s standard values. The average R^2^ for PT, H-A, and F-R models in typical hydrological years were 0.710, 0.703, and 0.748 in Baoding; 0.707, 0.718, and 0.746 in Xinji; and 0.644, 0.664, and 0.644 in Handan, respectively. All three models had R^2^>0.6, demonstrating their predictive effectiveness at the daily scale. However, the M-C model showed an average R^2^ of 0.500, 0.480, and 0.471 in Baoding, Xinji, and Handan, respectively, with R^2^<0.6, indicating a lower prediction effectiveness. Moreover, in each typical hydrological year, the F-R model consistently exhibits smaller RMSE and MAE compared with the PT and H-A models. Moreover, the WI is higher for the F-R model, indicating its superior predictive performance for daily ET_0_ values under varying hydrological conditions.

**Table 2:** Comparison the performances of different models under typical hydrological year at daily scale.

Hydrological year type	Evaporation model	Baoding				Xinji				Handan			
		R^2^	RMSE	MAE	WI	R^2^	RMSE	MAE	WI	R^2^	RMSE	MAE	WI
High flow year	PM	—	—	—		—	—	—		—	—	—	
	PT	0.637	1.886	1.554	0.670	0.659	2.082	1.659	0.698	0.626	2.385	1.784	0.672
	H-A	0.600	5.222	4.226	0.485	0.660	5.178	4.225	0.551	0.649	5.045	4.068	0.607
	M-C	0.359	2.115	1.714	0.731	0.415	2.073	1.668	0.774	0.455	2.107	1.666	0.803
	F-R	0.685	1.227	0.891	0.853	0.707	1.312	0.958	0.874	0.606	2.856	2.515	0.723
Median water year	PM	—	—	—		—	—	—		—	—	—	
	PT	0.755	2.483	1.839	0.680	0.722	2.099	1.705	0.691	0.612	2.611	1.957	0.632
	H-A	0.747	4.965	4.050	0.661	0.711	4.929	3.945	0.587	0.663	5.211	4.235	0.611
	M-C	0.566	2.056	1.649	0.856	0.500	2.262	1.792	0.795	0.553	2.028	1.633	0.848
	F-R	0.799	1.626	1.103	0.867	0.753	1.356	0.975	0.868	0.585	2.373	1.990	0.781
Low flow year	PM	—	—	—		—	—	—		—	—	—	
	PT	0.695	2.161	1.606	0.698	0.727	1.899	1.424	0.738	0.597	2.922	2.186	0.601
	H-A	0.715	5.249	4.293	0.598	0.749	5.334	4.454	0.573	0.623	4.739	3.803	0.651
	M-C	0.486	2.105	1.577	0.819	0.496	1.979	1.530	0.819	0.329	2.624	2.032	0.741
	F-R	0.724	1.441	0.990	0.867	0.768	1.166	0.787	0.908	0.632	2.151	1.820	0.821
Special dry year	PM	—	—	—		—	—	—		—	—	—	
	PT	0.754	2.721	2.052	0.669	0.719	2.174	1.665	0.700	0.742	2.473	1.931	0.684
	H-A	0.751	4.978	4.036	0.675	0.751	5.093	4.208	0.605	0.719	5.181	4.223	0.631
	M-C	0.591	2.231	1.736	0.859	0.511	2.052	1.600	0.827	0.547	2.259	1.746	0.836
	F-R	0.784	1.876	1.277	0.837	0.757	1.384	0.935	0.880	0.751	2.172	1.840	0.834

### 3.3 Comparative analysis of monthly ET_0_ values for different typical hydrologic years

**Figure 4:**
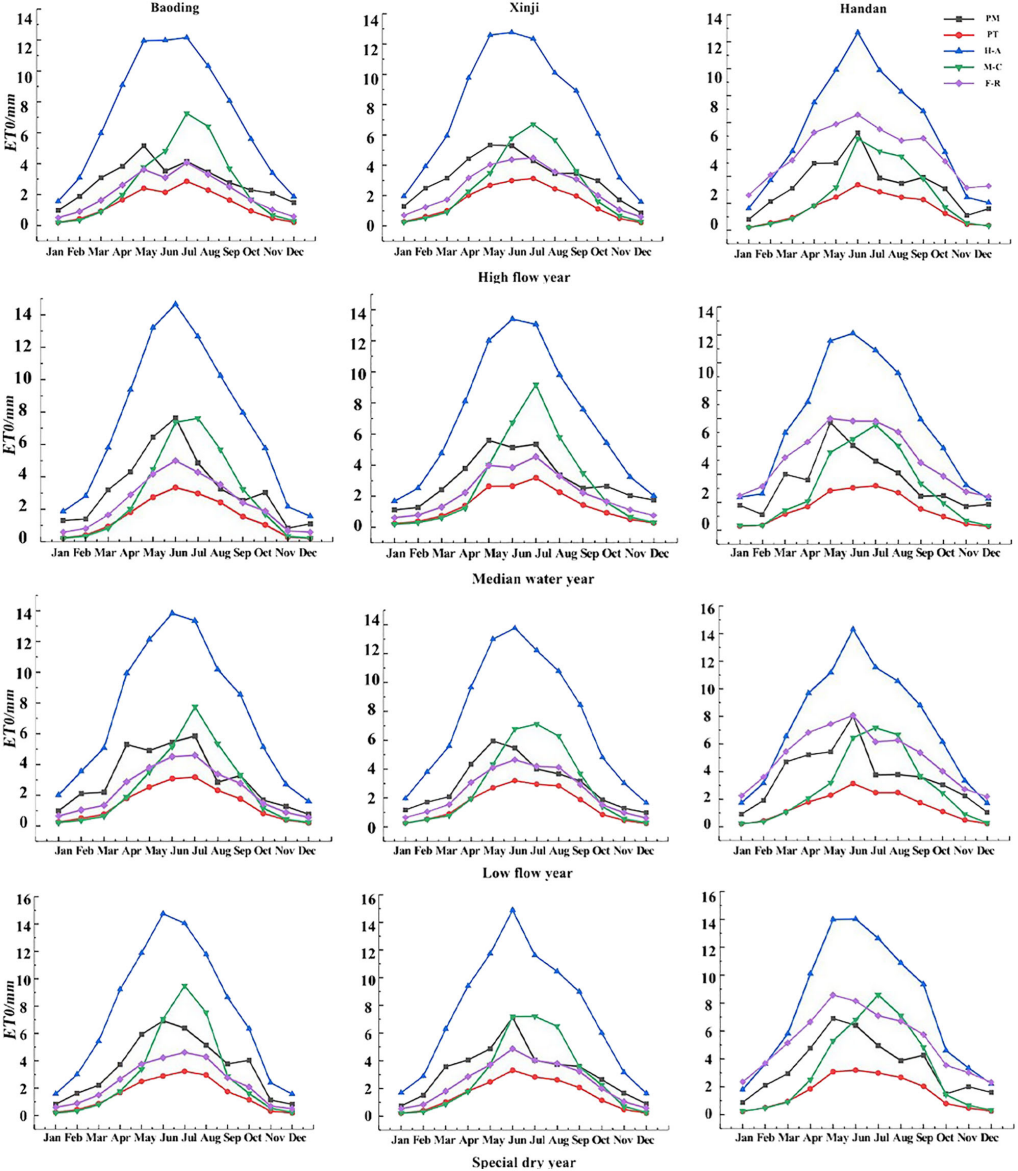
ET_0_ values of different models under typical hydrological year at monthly scale.

In **Figure 4**, the monthly ET_0_ trends of the five models across the three regions under various typical hydrological years exhibit patterns resembling monotonically increasing and decreasing parabolas. The upward segment spans from January to July, followed by a downward segment from July to December, with peak values occurring in the months of June and July for all five models. Similar to the daily trends, the H-A model consistently produces higher ET_0_ results than the PM model throughout the year. In contrast, the PT and F-R models consistently yield lower ET_0_ results than the PM model throughout the year. The M-C model produces higher ET_0_ results than the PM model in the months of June–September but lower values in the remaining months.

Using the PM model-calculated ET_0_ values as the standard, a comparative analysis of monthly ET_0_ values for the remaining four ET_0_ models is conducted under different typical hydrological years. At the monthly scale, the H-A model yielded slightly larger results than the PM model while the F-R and PT models produced slightly lower values. The remaining three models showed relatively close results to the PM model, except for the H-A model. The PM model and the other four models were used to calculate the RMSE, MAE, and R^2^ for each typical hydrological year. The results are presented in **Table 3**. At a significance level of 0.01, the PT, H-A, and F-R models exhibited good correlation with the PM model’s standard values. The average R^2^ for PT, H-A, and F-R models in typical hydrological years were 0.852, 0.900, and 0.879 for Baoding, Xinji, and Handan, respectively. All three models had R^2^ > 0.8, indicating better prediction effects at the monthly scale. However, the M-C model showed an average R^2^ of 0.622, 0.607, and 0.554 in Baoding, Xinji, and Handan, respectively, with R^2^ around 0.6, signifying poorer prediction compared with other models. Simultaneously, in each typical hydrological year, the F-R model consistently exhibits smaller RMSE and MAE compared with the PT and H-A models. Additionally, the WI is higher for the F-R model, indicating its superior predictive performance for monthly ET_0_ values under varying hydrological years.

**Table 3:** Comparison the performances of different models under typical hydrological year at monthly scale.

Hydrological year type	Evaporation Model	Baoding				Xinji				Handan			
		R^2^	RMSE	MAE	WI	R^2^	RMSE	MAE	WI	R^2^	RMSE	MAE	WI
High flow year	PM	—	—	—		—	—	—		—	—	—	
	PT	0.804	1.642	1.579	0.646	0.836	1.773	1.754	0.676	0.791	2.029	1.835	0.667
	H-A	0.855	5.065	4.198	0.406	0.923	4.982	4.196	0.471	0.870	4.820	4.026	0.550
	M-C	0.493	1.785	1.611	0.720	0.552	1.648	1.467	0.783	0.581	1.671	1.439	0.826
	F-R	0.840	0.905	0.768	0.864	0.865	0.893	0.773	0.901	0.892	2.399	2.311	0.705
Median water year	PM	—	—	—		—	—	—		—	—	—	
	PT	0.842	2.162	1.920	0.674	0.888	1.825	1.726	0.673	0.764	2.240	1.985	0.652
	H-A	0.886	4.816	4.014	0.614	0.919	4.757	3.886	0.530	0.831	5.002	4.207	0.566
	M-C	0.641	1.685	1.476	0.866	0.650	1.910	1.724	0.799	0.645	1.671	1.450	0.862
	F-R	0.874	1.271	0.994	0.881	0.909	0.998	0.883	0.887	0.822	1.883	1.660	0.811
Low flow year	PM	—	—	—		—	—	—		—	—	—	
	PT	0.839	1.842	1.692	0.711	0.851	1.612	1.485	0.743	0.729	2.513	2.314	0.611
	H-A	0.893	5.058	4.275	0.551	0.933	5.183	4.419	0.517	0.796	4.056	3.759	0.586
	M-C	0.608	1.608	1.294	0.848	0.621	1.645	1.417	0.836	0.369	2.164	1.823	0.746
	F-R	0.860	1.015	0.822	0.904	0.875	0.784	0.631	0.937	0.870	1.564	1.391	0.854
Special dry year	PM	—	—	—		—	—	—		—	—	—	
	PT	0.925	2.325	2.172	0.687	0.835	1.897	1.759	0.697	0.873	2.153	1.990	0.689
	H-A	0.964	4.772	4.006	0.635	0.922	4.932	4.192	0.546	0.932	4.955	4.188	0.580
	M-C	0.745	1.768	1.510	0.883	0.603	1.712	1.334	0.837	0.619	1.878	1.552	0.836
	F-R	0.942	1.398	1.173	0.867	0.861	1.029	0.780	0.902	0.943	1.807	1.727	0.832

### 3.4 Comparative analysis of 10-day ET_0_ values for different typical hydrologic years

**Figure 5:**
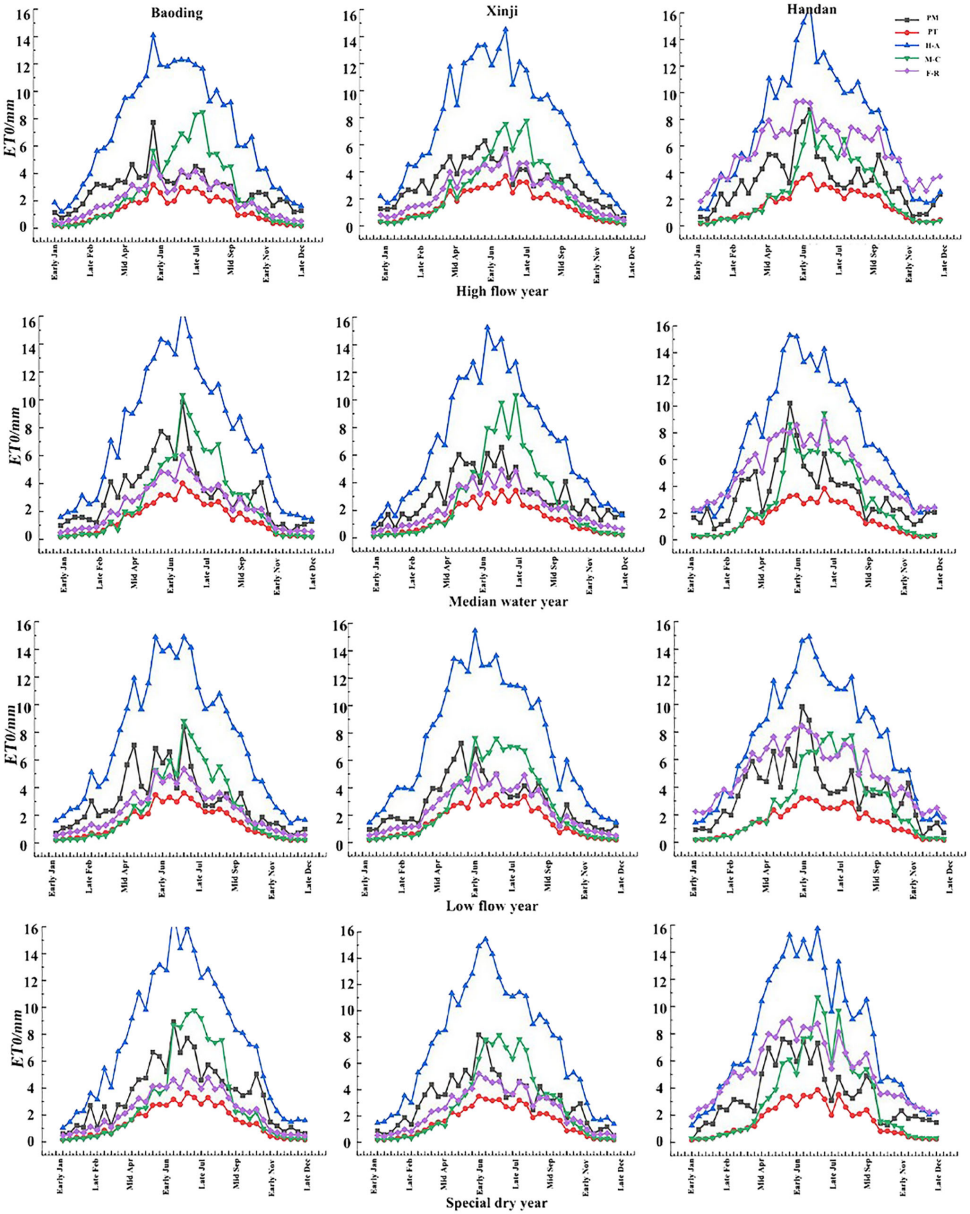
ET_0_ values of different models under typical hydrological year at the ten-day scale.

In **Figure 5**, the trends of 10-day ET_0_ values from the five models across the three regions under various typical hydrological years exhibit patterns resembling monotonically increasing and decreasing parabolas. The overall trend indicates an increase from January to around early July and a subsequent decrease from around early July to the end of December, with peak values occurring around early June to early July. Similar to the daily and monthly trends, the H-A model consistently produces higher ET_0_ results than the PM model throughout the year. In contrast, the PT and F-R models consistently yield lower ET_0_ results than the PM model throughout the year. The M-C model produces higher ET_0_ results than the PM model from mid-late June to early September, and the remaining 10-day ET_0_ values are lower than those of the PM model.

Using the PM model-calculated ET_0_ values as the standard, a comparative analysis of 10-day ET_0_ values for the remaining four ET_0_ models is conducted under different typical hydrological years. At the 10-day scale, the results of the H-A model are slightly larger than the standard value of the PM model, the results of the F-R model and the PT model are slightly lower than the standard value of the PM model, and the results of the other three models are relatively close to those of the PM model except for the H-A model. The PM model and the other four models were used to calculate the RMSE, MAE, and R^2^ for each typical hydrological year, and their corresponding results were analyzed. The results are shown in **Table 4**. At significance of 0.01, the analytical results of the three models, PT, H-A, and F-R, have good correlation with the standard values of the PM model, among which the average coefficients of determination (R^2^) of the three models, PT, H-A, and F-R, in typical hydrological years are 0.806, 0.835, and 0.840 in Baoding, Xinji, and Handan cities, respectively; 0.818, 0.885, and 0.851, respectively; and 0.743, 0.799, and 0.815 respectively. The R^2^ of the three models is >0.8, which proves that the three models have better prediction effects at the decadal scale. In contrast, the average coefficients of determination (R^2^) of the M-C model were 0.593, 0.560, and 0.521 in Baoding, Xinji, and Handan, respectively, with R^2^< 0.6, and the model predicted poorly. Meanwhile, during each typical hydrological year, the F-R model consistently demonstrates smaller RMSE and MAE in comparison with the PT and H-A models. Furthermore, the F-R model exhibits a higher WI, implying superior predictive accuracy for 10-day ET_0_ values across varying hydrological years.

**Table 4:** Comparison the performances of different models under typical hydrological year at 10-day scale.

Hydrological year type	Evaporation Model	Baoding				Xinji				Handan			
		R^2^	RMSE	MAE	WI	R^2^	RMSE	MAE	WI	R^2^	RMSE	MAE	WI
High flow year	PM	—	—	—		—	—	—		—	—	—	
	PT	0.744	1.700	1.549	0.936	0.897	1.806	1.661	0.934	0.752	2.120	1.781	0.919
	H-A	0.739	5.103	4.205	0.422	0.944	5.014	4.199	0.490	0.809	4.852	4.025	0.575
	M-C	0.466	1.875	1.617	0.729	0.718	1.748	1.559	0.777	0.522	1.823	1.584	0.814
	F-R	0.787	0.980	0.780	0.862	0.916	0.940	0.803	0.902	0.831	2.463	2.313	0.723
Median water year	PM	—	—	—		—	—	—		—	—	—	
	PT	0.822	2.239	1.836	0.920	0.840	1.887	1.708	0.930	0.711	2.342	1.946	0.910
	H-A	0.854	4.830	4.017	0.629	0.881	4.779	3.891	0.549	0.766	5.059	4.214	0.582
	M-C	0.634	1.770	1.520	0.868	0.597	2.022	1.751	0.794	0.625	1.735	1.521	0.863
	F-R	0.861	1.352	1.001	0.879	0.871	1.074	0.891	0.882	0.734	2.024	1.792	0.801
Low flow year	PM	—	—	—		—	—	—		—	—	—	
	PT	0.777	1.980	1.601	0.935	0.816	1.680	1.426	0.952	0.671	2.647	2.192	0.869
	H-A	0.814	5.095	4.273	0.571	0.885	5.215	4.417	0.533	0.739	4.548	3.759	0.614
	M-C	0.566	1.733	1.404	0.842	0.567	1.741	1.446	0.826	0.341	2.319	1.929	0.738
	F-R	0.809	1.190	0.869	0.886	0.848	0.872	0.634	0.931	0.789	1.715	1.557	0.846
Special dry year	PM	—	—	—		—	—	—		—	—	—	
	PT	0.882	2.416	2.048	0.912	0.809	0.352	1.671	0.932	0.835	2.238	1.937	0.921
	H-A	0.935	4.800	4.007	0.652	0.881	1.123	4.190	0.566	0.884	4.995	4.194	0.608
	M-C	0.705	1.877	1.591	0.877	0.562	0.021	1.497	0.830	0.596	2.016	1.634	0.835
	F-R	0.903	1.509	1.175	0.858	0.844	0.166	0.813	0.898	0.907	1.862	1.730	0.844

In conclusion, among the three models analyzed (PT, H-A, and F-R), all show predictive ability under different typical hydrological years, excluding the M-C model. The PT model demonstrates good correlation at daily, monthly, and 10-day scales across different regions. Specifically, the daily scale R^2^ in Baoding, Xinji, and Handan are 0.710, 0.707, and 0.644, respectively; the monthly scale R^2^ are 0.852, 0.852, and 0.789, respectively; and the 10-day scale R^2^ are 0.806, 0.818, and 0.743, respectively. The H-A model exhibits better correlation at different scales with daily scale R^2^ values in Baoding, Xinji, and Handan of 0.703, 0.718, and 0.664, respectively. The monthly scale R^2^ are 0.900, 0.924, and 0.857, while the 10-day scale R^2^ are 0.835, 0.885, and 0.799. However, the H-A model has larger RMSE and MAE values compared with the PT and F-R models, indicating higher prediction errors.

The F-R model shows good correlation at different scales with daily scale R^2^ values in Baoding, Xinji, and Handan of 0.748, 0.746, and 0.644, respectively. The monthly scale R^2^ are 0.879, 0.877, and 0.822, while the 10-day scale R^2^ are 0.840, 0.851, and 0.815. Compared with other models, the F-R model demonstrates higher R^2^ values, along with lower RMSE and MAE. Additionally, its WI is consistently higher across various time scales. Consequently, the F-R model shows superior applicability in the Haihe Plain region, particularly after correction, making it more suitable for predicting ET_0_ in this area.

### 3.5 FAO-24 Radiation improvement

The Bayesian estimation method is utilized to iteratively infer the empirical parameters a and b in the F-R model, leveraging meteorological data from Baoding City (1991–2014), Xinji City (2000–2016), and Handan City (1991–2014). The process entails computing posterior distributions of coefficient b using prior data, followed by iteratively calculating coefficient a by incorporating adjusted b values into the prior data. This iterative procedure refines model parameters, enhancing the accuracy of ET_0_ estimation. The specific procedure is as follows:

In accordance with the original F-R model, the two parameters can be expressed as:


(11) $$b=\frac{ET_{0}-a}{\frac{\text{Δ}}{\text{Δ}+\Upsilon }R_{s}}$$

(12) $$\;\;a=ET_{0}-b\frac{\text{Δ}}{\text{Δ}+\Upsilon }R_{s}$$

(2) The distribution of b and a values follows a normal distribution. The coefficient b was calibrated using Equations 11-13 using day-by-day meteorological data for a typical hydrological year.


(13) $$\;E=\frac{\alpha_{ο}\hat{\delta^{2}}+\hat{\Theta }0.81^{2}}{\hat{\delta^{2}}+0.81^{2}}$$

where E is the mathematical expectation, $\alpha_{ο}$ is the corresponding initial value, and $\;\hat{\text{Θ}}\;$ is the estimated mean as well as the variance $\hat{\delta^{2}}$ . Following the same procedure, the mathematical expectation of a is calculated by Equation 12 and Equation 13. The obtained expectations of parameters b and a are substituted into the F-R model in order to obtain the Calibrated F-R model as shown in **Table 5**.

**Table 5:** F-R correction model.

Study area	a	b	Calibrated F-R model
Baoding	0.15	0.74	$ET_{0}=0.15+0.74\left(\frac{\text{Δ}}{\text{Δ}+\text{Υ}}R_{s}\right)$
Xinji	0.13	0.69	$ET_{0}=0.13+0.69\left(\frac{\text{Δ}}{\text{Δ}+\text{Υ}}R_{s}\right)$
Handan	0.21	0.33	$ET_{0}=0.21+0.33\left(\frac{\text{Δ}}{\text{Δ}+\text{Υ}}R_{s}\right)$

### 3.6 Validation of improved F-R model

Following Shiri et al.’s recommendation (Shiri et al., 2015), validation with a distinct dataset was employed to ensure unbiased results. The original and calibrated models were evaluated using meteorological data from Baoding and Handan (2015–2019) and Xinji (2017–2021). ET_0_ values were computed for both monthly and 10-day periods derived from the daily values.

**Figure 6:**
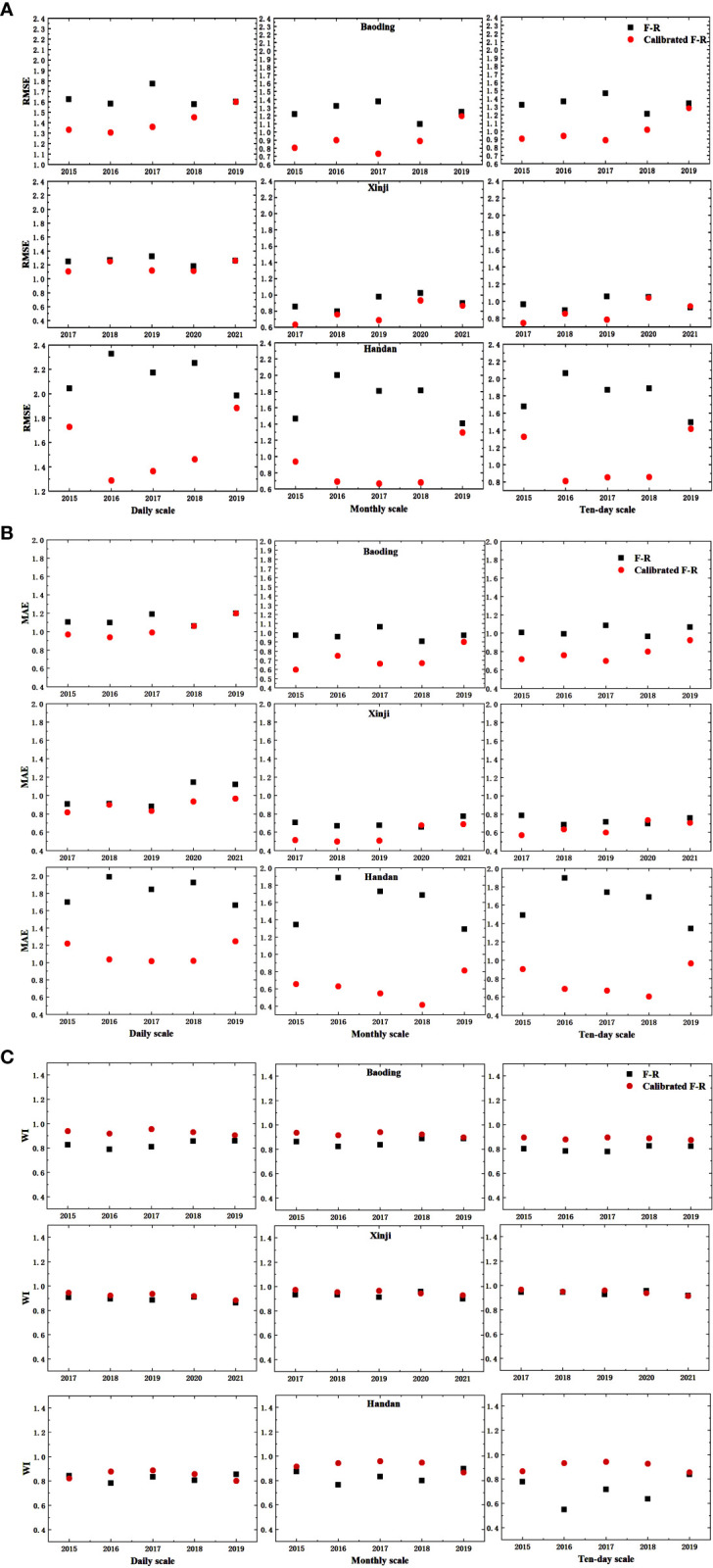
The comparisons of RMSE, MAE, and WI before and after the F-R model calibrated; **(A)** RMSE; **(B)** MAE; **(C)** WI.

After comparing the error analysis results in **Table 6** and **Table 7**, it is evident that under a significance level of P < 0.01, R^2^ remained unchanged. However, **Figure 6** shows significant decreases in RMSE and MAE across daily, monthly, and 10-day scales, accompanied by further improvements in WI. In Baoding City, Xinji City, and Handan City, the average coefficients of determination (R^2^) at the daily scale are 0.632, 0.746, and 0.693, respectively. At the monthly scale, the average R^2^ values are 0.769, 0.871, and 0.905, respectively, and at the 10-day scale, the average R^2^ values are 0.790, 0.838, and 0.852, respectively. There is good correlation at all three scales. Comparing the ET_0_ values before and after modification, the modified F-R model reduced RMSE by 15.81%, 29.51%, and 24.66% at the daily, monthly, and 10-day scales, respectively. MAE decreased by 19.04%, 34.47%, and 28.52% at the daily, monthly, and 10-day scales, respectively, while WI increased by 5.49%, 8.48%, and 10.78% at the daily, monthly, and 10-day scales, respectively. Therefore, the modified model can be effectively used for calculating reference crop evapotranspiration in the Haihe Plain region.

**Table 6:** Error analysis of the original F-R model.

Study area	Year	Daily scale				Monthly scale				Ten-day scale			
		R^2^	RMSE	MAE	WI	R^2^	RMSE	MAE	WI	R^2^	RMSE	MAE	WI
Baoding	2015	0.652	1.625	1.103	0.801	0.782	1.219	0.973	0.825	0.877	1.321	1.006	0.862
	2016	0.609	1.580	1.097	0.781	0.725	1.321	0.956	0.788	0.849	1.363	0.991	0.823
	2017	0.670	1.774	1.191	0.778	0.859	1.374	1.065	0.809	0.812	1.465	1.085	0.837
	2018	0.630	1.575	1.060	0.824	0.778	1.097	0.908	0.856	0.727	1.211	0.965	0.888
	2019	0.600	1.600	1.200	0.822	0.701	1.247	0.972	0.859	0.673	1.337	1.065	0.887
Xinji	2017	0.805	1.250	0.906	0.906	0.926	0.856	0.706	0.934	0.894	0.963	0.789	0.945
	2018	0.743	1.267	0.910	0.894	0.880	0.798	0.670	0.934	0.849	0.894	0.685	0.945
	2019	0.771	1.323	0.881	0.886	0.876	0.979	0.675	0.912	0.852	1.055	0.717	0.927
	2020	0.754	1.179	1.145	0.910	0.903	1.023	0.658	0.960	0.856	1.050	0.700	0.957
	2021	0.656	1.260	1.120	0.861	0.768	0.896	0.773	0.898	0.741	0.925	0.760	0.917
Handan	2015	0.675	2.043	1.699	0.842	0.909	1.465	1.345	0.874	0.810	1.677	1.490	0.778
	2016	0.693	2.327	1.989	0.782	0.885	1.999	1.884	0.765	0.848	2.062	1.895	0.548
	2017	0.751	2.172	1.845	0.834	0.943	1.807	1.727	0.832	0.907	1.869	1.741	0.715
	2018	0.664	2.252	1.923	0.804	0.890	1.814	1.685	0.800	0.838	1.886	1.688	0.637
	2019	0.681	1.985	1.660	0.853	0.896	1.408	1.290	0.897	0.856	1.492	1.345	0.837

**Table 7:** Error analysis of the calibrated F-R model.

Study area	Year	Daily scale				Monthly scale				Ten-day scale			
		R^2^	RMSE	MAE	WI	R^2^	RMSE	MAE	WI	R^2^	RMSE	MAE	WI
Baoding	2015	0.652	1.334	0.968	0.894	0.782	0.806	0.598	0.939	0.877	0.904	0.717	0.936
	2016	0.609	1.305	0.939	0.877	0.725	0.898	0.748	0.918	0.849	0.939	0.759	0.916
	2017	0.670	1.361	0.990	0.894	0.859	0.733	0.662	0.955	0.812	0.887	0.700	0.940
	2018	0.630	1.451	1.060	0.887	0.778	0.888	0.670	0.929	0.727	1.017	0.799	0.921
	2019	0.600	1.600	1.200	0.872	0.701	1.197	0.901	0.905	0.673	1.282	0.924	0.896
Xinji	2017	0.805	1.106	0.816	0.944	0.926	0.632	0.512	0.973	0.894	0.746	0.570	0.967
	2018	0.743	1.251	0.900	0.923	0.880	0.762	0.499	0.954	0.849	0.856	0.637	0.950
	2019	0.771	1.121	0.833	0.936	0.876	0.686	0.506	0.966	0.852	0.783	0.600	0.960
	2020	0.754	1.117	0.934	0.918	0.903	0.934	0.674	0.945	0.856	1.042	0.736	0.939
	2021	0.656	1.260	0.965	0.882	0.768	0.871	0.687	0.928	0.741	0.939	0.707	0.915
Handan	2015	0.675	1.728	1.218	0.822	0.909	0.939	0.656	0.914	0.810	1.324	0.904	0.862
	2016	0.693	1.288	1.036	0.876	0.885	0.691	0.629	0.943	0.848	0.810	0.689	0.930
	2017	0.751	1.365	1.014	0.886	0.943	0.667	0.547	0.958	0.907	0.854	0.668	0.941
	2018	0.664	1.462	1.019	0.857	0.890	0.680	0.415	0.947	0.838	0.856	0.605	0.925
	2019	0.681	1.884	1.244	0.801	0.896	1.296	0.811	0.866	0.856	1.416	0.967	0.853

## 4 Discussion

This study compared and evaluated the applicability of four evapotranspiration models—Priestley-Taylor (PT), Hargreaves (H-A), Mc-Cloud (M-C), and FAO-24 Radiation (F-R)—that use incomplete meteorological data. The Penman-Monteith (PM) model’s ET_0_ values were used as a benchmark for comparison. Overall, the simulation results highlight the superior performance of the F-R model among the four models.

The F-R model calculates ET_0_ mainly based on solar radiation data (Hauser, Gimon, Horin, & TX, 1999), which mainly uses the actual sunshine duration to obtain the solar magnetic declination, the atmospheric upper boundary solar radiation, and thus further the actual solar radiation. By analyzing the results of this study, under the condition of incomplete meteorological data, the simulated values of the F-R model at three scales of daily, monthly, and decadal under different typical hydrological years in three areas of the Haihe Plain before the modification have good correlation with the standard values of the PM model, and the results of the error analyses are also satisfactory. By further correcting the F-R model calculations, as shown in **Table 6** and **Table 7**, the R^2^ of the corrected F-R model did not change at a significance level of P < 0.01, whereas the RMSE and the MAE in the study area decreased substantially. Therefore, the predictions of the modified F-R model were more satisfactory and can be used for the calculation of ET of local reference crops.

The use of historical data for model calibration may lead to instability over time due to changing climate conditions. To address this, a suitable calibration method is essential. In this study, the simulated values of the Penman-Monteith (PM) model were employed as standards for the comparative analysis of four models: Priestly-Taylor (PT), Hargreaves (H-A), Mc-Cloud (M-C), and FAO-24 Radiation (F-R). The F-R model was identified as the most suitable for the Haihe Plain region with incomplete meteorological data. Considering geographical differences in the original F-R model’s applicability, a modification was performed using the Bayesian principle. This method utilizes known data as the prior distribution and recalculates data as the new posterior distribution, improving the reliability of the calculation by overcoming empirical data uncertainty and considering spatial-temporal variability. The Bayesian approach ensures a systematic and adaptive calibration method, enhancing stability and reliability in different scenarios. Beck et al. introduced Bayesian theory into model correction for the first time and clarified the basic idea of the correction (Beck and Katafygiotis, 1998), and also put forward a kind of adaptive MH algorithm-based Markov chain Monte Carlo method based on the MH algorithm (Beck and Au, 2002). Cheung et al. introduced and improved the hybrid Monte Carlo (HMCMC) method to solve the problem of Bayesian model correction for high-dimensional uncertainty parameters (Cheung and Beck, 2009). Currently, there is a recommended application of the modified Hargreaves model using Bayesian estimation method to calculate the ET of de-measured reference crops in the Sichuan Basin area (Feng et al., 2017). The modification of the F-R model using the Bayesian principle in the experimental area ensures the model’s applicability, providing a more accurate ET_0_ calculation. This enhanced model can serve as a scientific foundation for future farmland moisture management in the Haihe Plain area.

Different types of models have different sensitivities to meteorological data and are adapted to different regions. The PT model does not require wind speed and humidity data (Priestley and Taylor, 1972), and by comparing with the standard values of the PM model, the overall PT model simulation values are lower than those of the PM model, and the three scales of daily, monthly, and decadal are all well correlated under different typical hydrological years in the three regions of the Haihe Plain, and the error analysis. The results are relatively satisfactory. It can be used to calculate the ET_0_ in the Haihe Plain if the error is within the allowable range. In contrast to the PM model method for calculating reference ET, the PT model ignores the effect of water vapor deficit on reference ET, thus generating the assumption that ET_0_ depends only on solar radiation and temperature (Wu et al., 2021). This allows for PT modeling where PM modeling is not possible due to lack of data (Utset et al., 2004). It has been demonstrated that the simple and less data-demanding PT model is a good choice in many climatic regions (Jamieson, 1982; Pereira and Nova, 1992; Sau et al., 2004).

The M-C model is a simplified calculation method of ET_0_ based on temperature (McCloud, 1955), which just uses the daily mean temperature as meteorological data, and by comparing with the standard value of PM model, the correlation of this model is low, R^2^ < 0.6, indicating that this model is not good at predicting in the sea–river plain area. However, the M-C model is based on the daily mean air temperature, which is easy to calculate and especially suitable for areas with large differences in temperature variations (Valipour, 2015). The H-A model is suitable for the lack of radiative data and just uses the daily mean air temperature, daily maximum and daily minimum air temperature, and the atmospheric upper boundary solar radiation calculated through the daily ordinate. The simulated values of this model are compared with the PM model. The model simulated values are compared with the standard values of the PM model, and although there is a high correlation, the results of the error analysis are less satisfactory, with larger values of RMSE and MAE, and the model is not effective in predicting in the test area. However, many studies have confirmed that the H-A model is also a good predictor in some regions, and model optimization is continuously performed to better adapt to climate change (Gavilán et al., 2006; Tabari and Talaee, 2011; Berti et al., 2014; Cobaner et al., 2017). These calibrations are site-specific and cannot be extrapolated to some sites with completely different meteorological conditions.

This study warrants further validation, especially considering the absence of measured ET_0_ data. While the PM model served as the standard for calibrating the F-R model based on Bayesian theory, it is essential to verify the conclusions with measured data. Relying solely on model calculations, as highlighted by Martí et al (Martí et al., 2015), may yield unreasonable or incorrect conclusions. Therefore, incorporating measured ET_0_ data from lysimeters for calibration and evaluation is crucial. Moreover, while Bayesian theory allows for updating model parameters based on new sample data, it is important to note that this method is purely mathematical and overlooks the physical basis of the evapotranspiration process. Consequently, future research should emphasize calibrating the model using measured solar radiation data to enhance its accuracy.

## 5 Conclusions

In this study, we conducted a comparative analysis of four evapotranspiration models using incomplete meteorological data across various hydrological conditions to enhance ET_0_ estimation accuracy. The results revealed consistent spatial distribution trends among the models, with the F-R model demonstrating superior accuracy and predictive performance, particularly in terms of R^2^ and WI. Furthermore, the calibrated F-R model, refined through Bayesian theory, achieved higher accuracy, with R^2^ reaching 0.85 and WI reaching 0.9. The calibrated FAO-24 Radiation model offers valuable insights for precise ET_0_ estimation and irrigation decision-making in the Haihe Plain region, suggesting avenues for further accuracy improvements in future research.

## Data availability statement

The original contributions presented in the study are included in the article/supplementary material. Further inquiries can be directed to the corresponding authors.

## Author contributions

XS: Writing – review & editing, Writing – original draft, Visualization, Validation, Formal analysis, Data curation. BZ:Writing – original draft, Methodology, Data curation. MD:Visualization, Writing – original draft. RG: Writing – original draft, Visualization. CJ: Writing – original draft, Formal analysis. KM: Writing – original draft, Visualization. SG: Writing – original draft, Methodology. LG: Writing – review & editing, Supervision, Funding acquisition. WZ: Writing – review & editing, Supervision, Funding acquisition. XG: Methodology, Writing – review & editing, Supervision.
